# Supplementary material for: Sero-surveillance for IgG to SARS-CoV-2 at antenatal care clinics in three Kenyan referral hospitals: Repeated cross-sectional surveys 2020–21
Source: PLoS One. 2022 Oct 14;17(10):e0265478. doi: 10.1371/journal.pone.0265478 (PMC9565697; doi:10.1371/journal.pone.0265478)
Supplement: S1 Fig — (DOCX) [file pone.0265478.s001.docx]

## S1 Fig. Ante-natal care sample flow
