# Supplementary material for: Sero-surveillance for IgG to SARS-CoV-2 at antenatal care clinics in three Kenyan referral hospitals: Repeated cross-sectional surveys 2020–21
Source: PLoS One. 2022 Oct 14;17(10):e0265478. doi: 10.1371/journal.pone.0265478 (PMC9565697; doi:10.1371/journal.pone.0265478)

## S2 Fig. A) Daily (faint lines) and the 7-day moving average (bold lines) number of positive PCR tests per million population and B) Cumulative daily number of PCR positive tests for SARS-CoV-2 in Kenya, per million population


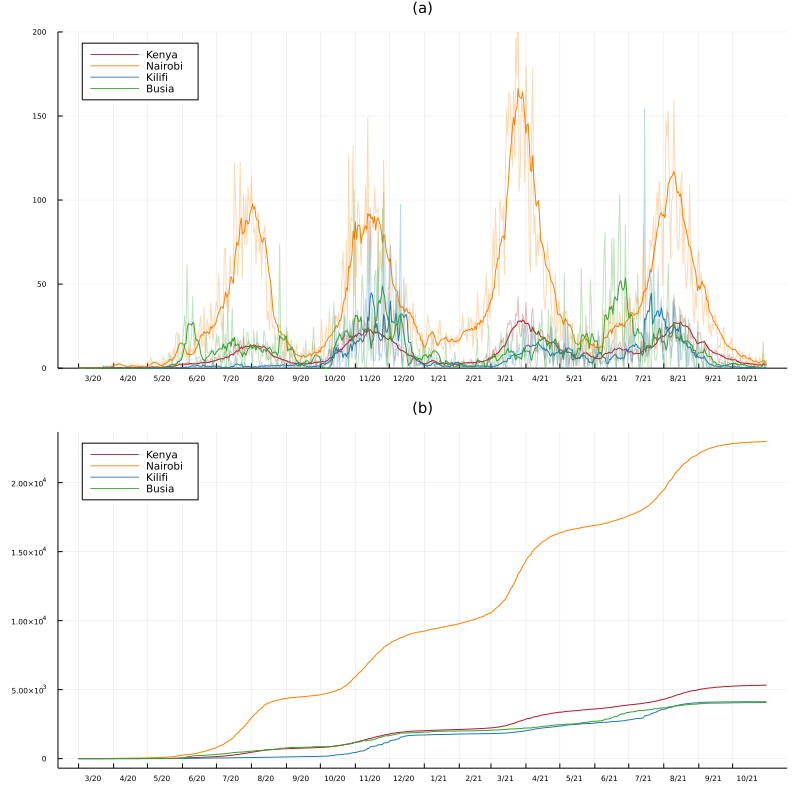

Supplement: S2 Fig — A) Daily (faint lines) and the 7-day moving average (bold lines) number of positive PCR tests per million population and B) Cumulative daily number of PCR positive tests for SARS-CoV-2 in Kenya, per million population. (DOCX) [file pone.0265478.s002.docx]
