# Supplementary material for: Sero-surveillance for IgG to SARS-CoV-2 at antenatal care clinics in three Kenyan referral hospitals: Repeated cross-sectional surveys 2020–21
Source: PLoS One. 2022 Oct 14;17(10):e0265478. doi: 10.1371/journal.pone.0265478 (PMC9565697; doi:10.1371/journal.pone.0265478)
Supplement: S1 Table — (A) Kenyatta National Hospital data (B) Busia Country Teaching & Referral Hospital. (DOCX) [file pone.0265478.s003.docx]

**Sero-surveillance for IgG to SARS-CoV-2 at antenatal care clinics in three Kenyan referral hospitals**

**Supplementary files**

**S1 Table. Comparability of the sample sets by round**

1. **Kenyatta National Hospital data, by round**

| **Kenyatta National Hospital, Nairobi** | | **Total** | **30^th^ July – 25^th^ August 2020** | | **27^th^ Jan- 11^th^ March 2021** | | **7^th^ September-19^th^ October 2021** | | **p-value** |
| --- | --- | --- | --- | --- | --- | --- | --- | --- | --- |
|  | | **N** | **n** | **%^1^** | **n** | **%^1^** | **n** | **%^1^** |  |
| All |  | 709 | 199 | - | 265 | - | 245 | - |  |
| Age | 17-29 years | 298 | 96 | (51.6) | 101 | (41.7) | 101 | (42.1) | 0.077 |
|  | 30-45 years | 370 | 90 | (48.4) | 141 | (58.3) | 139 | (57.9) |  |
| Trimester | 1 | 160 | 17 | (9.1) | 83 | (31.4) | 60 | (25.0) | <0.001 |
|  | 2 | 256 | 54 | (28.9) | 106 | (40.2) | 96 | (40.0) |  |
|  | 3 | 275 | 116 | (62.0) | 75 | (28.4) | 84 | (35.0) |  |
| Any symptoms in last month^1^ | Yes | 138 | 14 | (7.4) | 18 | (6.8) | 106 | (43.3) | <0.001 |
|  | No | 561 | 175 | (92.6) | 247 | (93.2) | 139 | (56.7) |  |
| Population density of sub-county of residence | <20,000 per km^2^ | 305 | 99 | (55.3) | 104 | (45.6) | 102 | (47.0) | 0.12 |
|  | 20-81,000 per km^2^ | 319 | 80 | (44.7) | 124 | (54.4) | 115 | (53.0) |  |

^1^Column percentages

1. **Busia County Teaching & Referral Hospital data, by round**

| **Busia County Teaching & Referral Hospital** | | **Total** | **15^th^ April - 21 May 2021** | | **20 September – 22 October 2021** | | **p-value** |
| --- | --- | --- | --- | --- | --- | --- | --- |
|  |  | **N** | **n** | **%** | **n** | **%** |  |
| All |  | 567 | 270 | - | 297 | - |  |
| Age | 17-29 years | 217 | 14 | (66.7) | 203 | (70.7) | 0.69 |
|  | 30-45 years | 91 | 7 | (33.3) | 84 | (29.3) |  |
| Trimester | 1 | 129 | 51 | (19.3) | 78 | (26.7) | 0.007 |
|  | 2 | 367 | 192 | (72.5) | 175 | (59.9) |  |
|  | 3 | 61 | 22 | (8.3) | 39 | (13.4) |  |
| Any symptoms in last month^1^ | Yes | 274 | 109 | (40.4) | 165 | (56.1) | <0.001 |
|  | No | 290 | 161 | (59.6) | 129 | (43.9) |  |
