# Supplementary material for: Sero-surveillance for IgG to SARS-CoV-2 at antenatal care clinics in three Kenyan referral hospitals: Repeated cross-sectional surveys 2020–21
Source: PLoS One. 2022 Oct 14;17(10):e0265478. doi: 10.1371/journal.pone.0265478 (PMC9565697; doi:10.1371/journal.pone.0265478)
Supplement: S2 Table — (DOCX) [file pone.0265478.s004.docx]

**S2 Table. Median OD ratios among unvaccinated, seropositive participants over time**

| **Location** | **Timing** | **Median OD ratio (IQR) among unvaccinated** | | **Median OD ratio (IQR) among unvaccinated, ‘threshold’ seropositive** | |  |
| --- | --- | --- | --- | --- | --- | --- |
| KNH | | |  | |  | |
|  | Aug 2020 | 1.79 (0.95-3.40) | | 3.48 (2.9-5.3) | |  |
|  | Feb 2021 | 1.05 (0.82-2.23) | | 3.22 (2.6-4.7) | |  |
|  | Oct 2021 | 6.48 (2.83-14.1) | | 8.23 (5.8-17.0) | |  |
| BCTRH | | |  | |  | |
|  | May 2021 | 1.29 (0.96-2.32) | | 3.00 (2.5-4.0) | |  |
|  | October 2021 | 3.67 (1.04-7.69) | | 6.34 (3.8-11.0) | |  |
| KCH | | |  | |  | |
|  | Oct 2020 | 0.65 (0.56-0.80) | | 2.94 (2.2-4.0) | |  |
|  | Nov 2020 | 0.88 (0.75-1.14) | | 3.55 (3.1-8.3) | |  |
|  | Dec 2020 | 1.07 (0.87-1.84) | | 4.47 (2.2-6.8) | |  |
|  | Mar ‘21 | 0.99 (083-1.79) | | 3.42 (2.9-4.8) | |  |
|  | Apr ‘21 | 1.06 (0.82-1.73) | | 3.52 (2.5-4.7) | |  |
|  | May ‘21 | 1.13 (0.87-2.24) | | 3.14 (2.5-4.4) | |  |
|  | June ‘21 | 0.99 (0.86-1.94) | | 3.89 (2.5-7.5) | |  |
|  | July ‘21 | 2.15 (0.69-4.65) | | 4.63 (2.8-7.3) | |  |
|  | Aug ‘21 | 3.04 (0.79-8.42) | | 7.34 (3.6-12.9) | |  |
|  | Sept ’21 | 2.56 (1.06-5.87) | | 5.07 (3.4-8.2) | |  |
|  | Oct ‘21 | 2.75 (0.82-6.04) | | 5.83 (4.0-8.7) | |  |
