# Supplementary material for: Sero-surveillance for IgG to SARS-CoV-2 at antenatal care clinics in three Kenyan referral hospitals: Repeated cross-sectional surveys 2020–21
Source: PLoS One. 2022 Oct 14;17(10):e0265478. doi: 10.1371/journal.pone.0265478 (PMC9565697; doi:10.1371/journal.pone.0265478)
Supplement: S1 Appendix — (DOCX) [file pone.0265478.s005.docx]

**Statistical appendix: Stan code and input data for estimating adjusted seroprevalence**

data {

int N;

int N_se;

int N_sp;

int y;

int x;

int z;

}

parameters {

real<lower=0,upper=1> p;

real<lower=0,upper=1> se;

real<lower=0,upper=1> sp;

}

transformed parameters {

real<lower=0,upper=1> p_obs;

p_obs = se * p + (1 - sp) * (1 - p);

}

model {

//priors

p ~ beta(1, 1);

se ~ beta(1, 1);

sp ~ beta(1, 1);

//likelihood

y ~ binomial(N, p_obs);

x ~ binomial(N_se, se);

z ~ binomial(N_sp, sp);

}

**Data:**

|  | **NBO overall** | **NBO 17-29y** | **NBO 30-45y** | **NBO Tri 1** | **NBO Tri 2** | **NBO Tri 3** | **NBO symptoms present** | **NBO**  **No symptoms** | **NBO pop density**  **<20k** | **NBO pop density**  **>20k** | **Kilifi Sept** | **Kilifi Oct** | **Kilifi Nov** |
| --- | --- | --- | --- | --- | --- | --- | --- | --- | --- | --- | --- | --- | --- |
| y | 91 | 38 | 44 | 7 | 21 | 58 | 7 | 78 | 44 | 39 | 0 | 3 | 16 |
| x | 166 | 166 | 166 | 166 | 166 | 166 | 166 | 166 | 166 | 166 | 166 | 166 | 166 |
| z | 901 | 901 | 901 | 901 | 901 | 901 | 901 | 901 | 901 | 901 | 901 | 901 | 901 |
| N | 196 | 91 | 90 | 17 | 53 | 114 | 12 | 172 | 97 | 79 | 82 | 183 | 154 |
| N_se | 179 | 179 | 179 | 179 | 179 | 179 | 179 | 179 | 179 | 179 | 179 | 179 | 179 |
| N_sp | 910 | 910 | 910 | 910 | 910 | 910 | 910 | 910 | 910 | 910 | 910 | 910 | 910 |

The distribution of OD ratios in unvaccinated, *seropositive Nairobi* samples, by round, indicating the potential for natural boosting to have occurred (The seropositivity threshold is log(2) = 0.69 on the log scale)

The distribution of OD ratios in unvaccinated Busia samples, by batch, indicating the potential for natural boosting to have occurred (The seropositivity threshold is log(2) = 0.69 on the log scale)
