# Supplementary material for: Sero-surveillance for IgG to SARS-CoV-2 at antenatal care clinics in three Kenyan referral hospitals: Repeated cross-sectional surveys 2020–21
Source: PLoS One. 2022 Oct 14;17(10):e0265478. doi: 10.1371/journal.pone.0265478 (PMC9565697; doi:10.1371/journal.pone.0265478)
Supplement: Attachment — Submitted filename: Reviewer comments on PONE-D-22-06158.docx [file pone.0265478.s006.docx]

**Reviewer comments on the manuscript entitled “Sero-surveillance for IgG to SARS-CoV-2 at antenatal care clinics in three Kenyan referral hospitals: repeated cross-sectional surveys 2020-21” (Manuscript ID PONE-D-22-06158)**

The authors of this manuscript, entitled “Sero-surveillance for IgG to SARS-CoV-2 at antenatal care clinics in three Kenyan referral hospitals: repeated cross-sectional surveys 2020-21”, presented a cross-sectional study aimed at assessing the transmission of SARS-CoV-2 in Kenya. The manuscript fits the aim of “PLOS ONE,” which publishes papers and research in the area of the natural sciences, medical research, engineering, and the related social sciences and humanities.

The title, as well as the abstract, are informative; nevertheless, the study’s aim has not been clearly described and needs to be elucidated in more detail,

Introduction requires a deeper contextualization of SARS-CoV-2 infection

Finally, the text should be written in a scientific style containing passive voice and 3rd person.

Moreover, check the references list and the references cited in the text. Please review them for completeness and errors and note that according to PLOS ONE, the references must be cited in the Vancouver style.

Therefore, I strongly recommend accepting the manuscript after minor revision.

Prof. Veronica Papa, Ph.D

Department of Motor Sciences and wellness,

University of Naples “Parthenope”

veronica.papa@uniparthenope.it
